# Setting of Plaster of Paris

**Published:** 1899-07

**Authors:** J. A. Belcher


### Setting of Plaster of Paris.

BY J. A. BELCHER.

As the results of experiments undertaken to determine the effect of various agents on the setting of plaster of Paris, found that two drachms of plaster, mixed with one drachm of a 5 percent solution of sodium chloride, hardened in two minutes. Mixed with one drachm of a 5 percent solution of sugar, it hardened in three and a half minutes. Mixed with one drachm of a 1 percent sodium chloride solution, it hardened in five minutes. Mixed with one drachm of an 0.5 percent sodium chloride solution, it hardened in five minutes. Mixed with one drachm of a 5 percent calcium chloride solution, it hardened in six and a half minutes. Mixed with one drachm of tap water, it hardened in nine minutes. Mixed with one drachm of distilled water, it hardened in nine minutes. Mixed with one drachm of saturated solution of sodium chloride it hardened in eighteen minutes. Mixed with one drachm of a 5 percent solution of glycerine in distilled water, it hardened in nineteen minutes. Mixedwith one drachm of a 5 percent solution of white of egg in distilled water, it hardened in twenty minutes. Mixed with one drachm of a 10 percent solution of white of egg in distilled water, it hardened in twenty-five minutes. Mixed with one drachm of a 10 percent solution of glycerine in distilled water, it hardened in thirty-five minutes. Mixed with one drachm of a 25 percent solution of glycerine in distilled water, it hardened in sixty minutes. It would appear that where it is of importance to make plaster of Paris set rapidly it should be mixed with a 5 percent solution of common salt, such as may be made roughly by adding a tablespoonful of salt to a pint of water.—*Treatment.*

